# Kawasaki disease presenting after intussusception: a case report

**DOI:** 10.1186/s13256-021-02942-y

**Published:** 2021-06-23

**Authors:** Rukhsar Shabir Osman, Hajaj Mohamed Salum, Mariam Noorani

**Affiliations:** 1 Aga Khan Hospital, Dar es Salaam, Tanzania; 2 Muhimbili University of Health and Allied Science, Dar es Salaam, Tanzania

**Keywords:** Intussusception, Kawasaki, Tanzania, Fever

## Abstract

**Introduction:**

Kawasaki disease is a common vasculitis of unknown etiology that occurs mainly in preschool children. It manifests as a self-limited acute febrile illness with other features including extremity changes, cervical lymphadenopathy, oropharyngeal changes, truncal rash, and conjunctivitis. Intestinal involvement is not uncommon, with abdominal pain and vomiting being the most frequently reported symptoms. Intussusception has been described as a manifestation or complication of this disease, but few reports exist in literature.

**Case presentation:**

A 7-month-old boy of Asian origin who presented with vomiting and passage of bloody mucoid stool was diagnosed with intussusception that was successfully reduced during emergency laparotomy. The baby was discharged home post-surgery following clinical improvement. He was readmitted on the fourth postoperative day with fever, irritability, and diarrhea. He was investigated and treated for presumed intraabdominal sepsis with multiple antibiotics with no improvement. He gradually developed the mucocutaneous features of Kawasaki disease and was treated with intravenous immunoglobulin and aspirin with good outcome.

**Conclusion:**

Intussusception and Kawasaki disease both commonly occur in children less than 2 years old. It is important to include Kawasaki disease as a differential diagnosis in children of this age who present with an acute febrile illness and gastrointestinal symptoms. A common underlying pathologic process could be contributing to both conditions.

## Background

Kawasaki disease (KD) is an acute febrile vasculitic syndrome of childhood. It was first reported by its namesake, Dr. Tomisaku Kawasaki, in 1967 in Japan. It has been noted in all ethnic origins, with highest incidence being reported among children of Asian origin [1]. Despite sporadic cases being reported across many countries in Africa, particularly North and West African regions, the epidemiological data for KD are limited for African countries [2].

A disease once thought to be benign and self-limited is actually a leading cause of acquired heart disease among children in developed countries and a risk factor for adult ischemic heart disease [1] .

The cause of this disease is to date unknown; however, multiple theories have been proposed, ranging from genetic and environmental factors to infectious etiology [3] .

Kawasaki disease has been categorized into complete (typical) and incomplete (atypical) disease. Diagnostic criteria for typical disease are fever for at least 5 days without an explanation, plus any four of the following five: bilateral bulbar conjunctival congestion; polymorphous rash; acute nonpurulent cervical lymphadenopathy; changes of the lips and oral cavity (red or fissured lips, strawberry tongue, diffuse injection of pharyngeal mucosa); or peripheral extremity changes including erythema of palms or soles, edema of hands or feet (acute phase), and periungual desquamation (convalescent phase). The characteristic features can be preceded by nonspecific gastrointestinal or respiratory symptoms such as diarrhea, vomiting, abdominal pain, decreased intake, irritability or cough, and rhinorrhea. Diagnosis can be delayed in those initially presenting with gastrointestinal symptoms [3].

A high index of suspicion is necessary to identify cases of Kawasaki disease and initiate early treatment, since children in sub-Saharan Africa suffer from many tropical and infectious diseases that present with symptoms similar to those of Kawasaki disease such as fever, irritability, and a rash. These include malaria, typhoid fever, meningitis, viral exanthem such as measles and roseola infantum, and infection with group A beta hemolytic streptococcus [2].

This is a report of a patient with Kawasaki disease associated with intussusception who was admitted at our hospital.

## Case report

A 7-month-old fully vaccinated Asian boy was presented with 1 day history of nonbilious, nonprojectile vomiting, and passage of bloody mucoid stool (Table 1). There was no history of fever or upper respiratory infection. He had been introduced to complementary feeding consisting of cow’s milk and formula feeding, 2 weeks prior to illness.

**Table 1:** Time line of events

Date	Summary from initial and follow-up visits	Diagnostic testing	Intervention
April 2019 Day 1	One-day history of vomiting and mucoid bloody stool. On examination, gloved finger stained with mucoid feces mixed with streaks of fresh blood. Diagnosis: possible intussusception	CRP 3.05 mg/L, WBC: 16.13 × 10^9^/L, platelets: 466 × 10^9^/L, Hb: 10.7 g/dL, abdominal ultrasound: target sign at right upper quadrant with peripheral vascularity	Laparotomy and manual reduction of the intussusception. Initiation of ceftriaxone and metronidazole
Day 3	Child doing well		Discharged on cefixime and metronidazole
Day 4	High-grade fevers and diarrhea. Examination: irritable, febrile, tachycardic with some dehydration, no lymphadenopathy, delayed capillary refill with peripheral cyanosis, and maculopapular rash on the back and at BCG scar site. Hyperactive bowel sounds and clean surgical wound. Diagnosis: sepsis	CRP: 121 mg/L, WBC: 16.83 × 10, platelets: 428 × 10^9^/L, Hb: 9.1 g/dL ^9^/L, stool routine, urine routine: normal Abdominal X-ray: normal	IV amikacin + IV ceftriaxone + IV metronidazole IV fluid bolus, then IV DNS at maintenance rate, zinc sulfate 20 mg, ibuprofen, and paracetamol
Day 5	Persistent fever and irritability, nonpitting feet swelling, bilateral nonpurulent conjunctivitis sparing the limbus	Dengue serology: negative	Antibiotics continued
Day 6	Persistent fever erythematous change of the tongue, dry cracked and bleeding lips. Diagnosis: Kawasaki disease	WBC: 8 × 10^9^/L, platelets: 316 × 10^9^/L, Hb: 8.6 g/dL CRP: 182 mg/L Echocardiogram: normal	IV immunoglobulin at 2 g/kg and low-dose aspirin at 3 mg/kg/day
Day 7	Fever resolved		Discharged on low-dose aspirin
May 2019	Follow-up visit: no signs and symptoms	Normal echocardiogram and CRP	Aspirin stopped

CRP: c-reactive protein, WBC: white blood cells, Hb: hemoglobin, IV: intravenous, DNS: dextrose normal saline

He was delivered at 36 weeks gestation age to a mother with gestational diabetes, with an Apgar score of 9^1^ and 10^5^, and required continuous positive airway pressure (CPAP) at birth because of respiratory distress.

On examination, he was irritable with dry oral mucosa, warm extremities with capillary refill < 2 seconds, pulse rate 133 beats per minute, and respiratory rate 40 breaths per minute. His throat was not hyperemic, and tympanic membrane was clear, lung fields were clear with vesicular breath sounds bilaterally, no added sounds were heard on auscultation of the heart, and in the abdomen no tenderness or palpable mass was felt. On anal examination, there was no obvious fissure, and on digital examination, there was no palpable mass and gloved finger was stained with mucoid feces mixed with streaks of fresh blood.

**Fig. 1:**
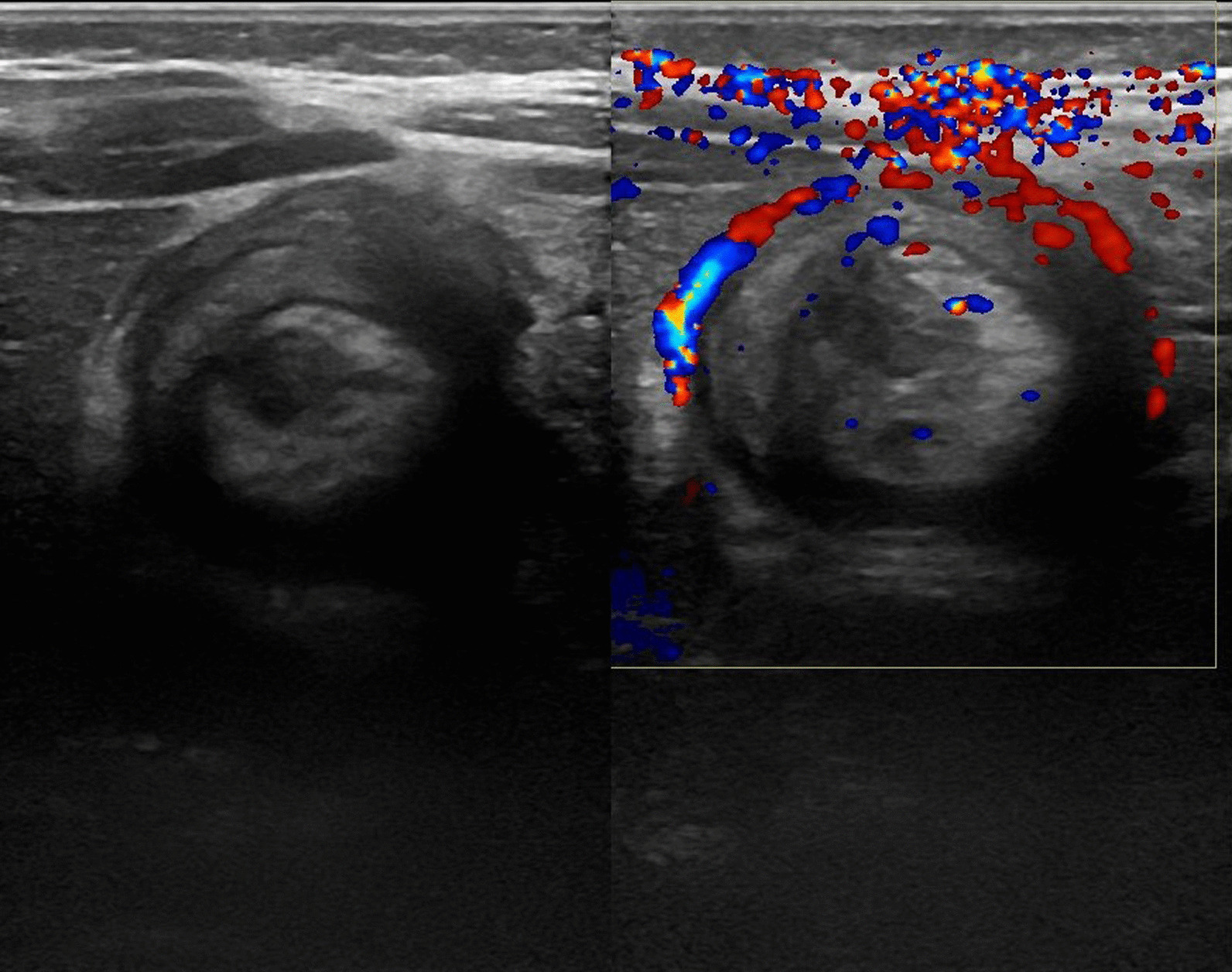
Ultrasound showing target sign with peripheral vascularity

A provisional diagnosis of intussusception was made and confirmed on abdominal ultrasound, which showed a target sign at the right upper quadrant with peripheral vascularity, highly suspicious of intussusception (Fig. 1). Laboratory tests showed initial C-reactive protein (CRP) 3.05 mg/L, white blood cells (WBC) 16.13 × 10^9^ /L, neutrophils 64.4%, lymphocytes 30.8%, platelets 466 × 10^9^/L, and hemoglobin 10.7 g/dL. Laparotomy was performed with intraoperative findings of ileocolic intussusception with viable bowel, and manual reduction was done. The child was started on intravenous ceftriaxone 75 mg/kg/day and intravenous metronidazole 30 mg/kg/day. He had an uneventful postoperative period and was discharged 2 days later on oral antibiotics.

The child presented again on the fourth day post-laparotomy after having developed high-grade fevers associated with several episodes of watery yellowish, nonbloody, nonmucoid stools, and skin rash on the back that started at the site of bacillus Calmette–Guerin (BCG) scar on the right arm. On examination, he was irritable and febrile with temperature of 39.9 °C, and had no lymphadenopathy, no pallor but some dehydration, tachycardia (163 beats per minute), and respiratory rate of 46 breaths per minute. He had peripheral cyanosis with capillary refill of about 4 seconds. There was a hyperemic maculopapular rash on the back and at the BCG vaccination site. His abdomen was soft, nontender, and not distended. He had hyperactive bowel sounds with a clean surgical wound.

Workup was done that showed CRP 121 mg/L, WBC 16.83 × 10^9^/L, neutrophils 54%, lymphocytes 33.9%, platelets 428 × 10^9^/L, hemoglobin 9.1 g/dL, normal stool routine [no red blood cells (RBC), leukocytes, cysts, or ova], and normal urine routine (no pus cells or RBC, negative nitrites), and blood culture was done in the following manner: 1 ml of blood was collected aseptically in a BD BACTEC bottle and cultured for 7 days using an automated blood culture system and using fluorescent technology to detect growth; however, no growth was detected. An abdominal X-ray was obtained, which showed no free gas in the abdomen, and abdominal ultrasound showed no fluid collection with normal bowel peristalsis. A diagnosis of presumed intraabdominal sepsis was made, and intravenous amikacin at 15 mg/kg/day was added to metronidazole and ceftriaxone. Normal saline bolus 160 ml was administered, after which perfusion improved. He was then maintained on intravenous dextrose saline, zinc sulfate 20 mg once a day for diarrhea, and oral ibuprofen 10 mg/kg/dose and oral paracetamol 15 mg/kg/dose for fever control. On the fifth day post-surgery, fever and irritability persisted, and the child now developed swelling of both feet and bilateral nonpurulent conjunctivitis sparing the limbus. Serology for dengue was done, which was negative. On day 6 post-laparotomy, erythematous change of the tongue was noticed with dry cracked and bleeding lips. The fever was persistent, and a repeat workup was done: CRP 182 mg/L, WBC 8 × 10^9^/L, platelets 316 × 10^9^/L, and hemoglobin 8.6 g/dL. A diagnosis of Kawasaki disease was made, which met four out of the five criteria (rash, eye changes, mucous membrane changes, extremity changes), and intravenous immunoglobulin at 2 g/kg was given with low-dose oral aspirin at 3 mg/kg/day. An echocardiogram was done, which found normal coronary arteries. Within 24 hours of the intravenous immunoglobulin (IVIG), the child was fever free and was discharged the following day on low-dose aspirin. On follow-up 48 hours later, CRP was 34 mg/L and swelling of hands and feet had resolved. He was scheduled for cardiology follow-up, which was normal.

## Discussion

Gastrointestinal presentation such as abdominal pain, vomiting and diarrhea occurs in 25–30% of Kawasaki cases [4]. This can cause diagnostic delay, especially when diagnostic criteria are lacking during the febrile phase. In an Italian study, 4.6% of patients with Kawasaki disease presented with acute surgical abdomen [5], which included hydrops of the gall bladder, ischemic colitis, mesenteric and splenic ischemia, hemorrhagic duodenitis, appendicular involvement, and intussusception.

There are four reported cases of Kawasaki disease associated with intussusceptions in literature, of which two presented with intussusception after diagnosis of Kawasaki disease: a 3-year-old girl with ileocolic intussusception [6] and a 4-month-old girl with ileocecal intussusception [7]. The other two presented with intussusception prior to diagnosis of Kawasaki disease: a 3-year-old boy with colocolic intussusception that was later recognized as Kawasaki disease [8] and a 3-month-old boy [8].

In our case, the child first presented to the hospital with acute onset of vomiting and bloody mucoid stool without fever or other Kawasaki diagnostic criteria features; hence, he was diagnosed with intussusception. On subsequent admission, he presented with peripheral cyanosis and capillary refill time of 4 seconds, which needed fluid boluses for correction, very high CRP (121 mg/L), and platelets at the higher end of normal range (428 × 10^9^/L) with a history of laparotomy a week prior, leading us to our initial diagnosis of sepsis. However, negative imaging tests, nonresponse of fever, overall clinical condition, increasing C-reactive protein in spite of being on three different antibiotics, and development of the mucocutaneous features of Kawasaki disease confirms that this was not sepsis.

A relationship between gastrointestinal (GI) symptoms and Kawasaki disease has been hypothesized by Yamashiro, who reported GI tract as one of the primary entry portal for bacterial toxins that affect the immune system by acting as superantigens in Kawasaki disease [9].

The pathogenesis of intussusception depends on the underlying causes, which include lead point (Meckel’s diverticulum, polyp, duplication cyst, tumor, hematoma, or vascular malformation). Infections, both viral and bacterial, can stimulate lymphatic tissue in the intestinal tract, resulting in hypertrophy of Peyer’s patches in the lymphoid-rich terminal ileum, which may act as a lead point for ileocolic intussusception. Similarly, Kawasaki disease is associated with inflammatory changes of the bowel wall and vasculitis, which could act as a lead point for intussusception.

Kawasaki disease has been reported fewer times in Africa compared with Asia, America, and Europe [2].This is the third case of Kawasaki disease to be reported in Tanzania and probably the first to present with intussusception. There is likelihood that the two conditions are associated; KD could have resulted in vasculitis and aneurysm of medium-sized submucosal vessel of the ileum or a hypertrophied Peyer’s patch that could have acted as a lead point for intussusception.

## Conclusion

It is very important to consider Kawasaki disease in children who are diagnosed with intussusception presenting with or followed by a febrile illness. Our case serves as a reminder that Kawasaki disease may present variably, and not every fever associated with a surgical abdomen is sepsis.

## Data Availability

Not applicable.

## Abbreviations

KD: Kawasaki disease
IVIG: Intravenous immunoglobulin
CRP: C-reactive protein
WBC: White blood cells
IV: Intravenous
BCG: Bacillus Calmette–Guerin
RBC: Red blood cell
DNS: Dextrose normal saline
GI: Gastrointestinal
